# The Deep Rocky Biosphere: New Geomicrobiological Insights and Prospects

**DOI:** 10.3389/fmicb.2021.785743

**Published:** 2021-11-30

**Authors:** Hinako Takamiya, Mariko Kouduka, Yohey Suzuki

**Affiliations:** Department of Earth and Planetary Science, The University of Tokyo, Bunkyo, Japan

**Keywords:** subsurface microbiology, deep rocky habitats, extremophile habitability, astrobiology, omics-based evo-phylogeny, ecophysiology, deep biosphere

## Abstract

Rocks that react with liquid water are widespread but spatiotemporally limited throughout the solar system, except for Earth. Rock-forming minerals with high iron content and accessory minerals with high amounts of radioactive elements are essential to support rock-hosted microbial life by supplying organics, molecular hydrogen, and/or oxidants. Recent technological advances have broadened our understanding of the rocky biosphere, where microbial inhabitation appears to be difficult without nutrient and energy inputs from minerals. In particular, microbial proliferation in igneous rock basements has been revealed using innovative geomicrobiological techniques. These recent findings have dramatically changed our perspective on the nature and the extent of microbial life in the rocky biosphere, microbial interactions with minerals, and the influence of external factors on habitability. This study aimed to gather information from scientific and/or technological innovations, such as omics-based and single-cell level characterizations, targeting deep rocky habitats of organisms with minimal dependence on photosynthesis. By synthesizing pieces of rock-hosted life, we can explore the evo-phylogeny and ecophysiology of microbial life on Earth and the life’s potential on other planetary bodies.

## Introduction

The discovery of deep-sea hydrothermal vents has dramatically changed our perspective on life (Corliss et al., 1979). The deep-sea hydrothermal vent, which is densely colonized by peculiar organisms, such as tubeworms and giant clams around black smoker chimneys, is known to be devoid of nutrients from photosynthesis; instead, nutritional dependence is mainly on chemicals emitted from the black smokers (Felbeck, 1981). Chemosynthesis, a term commonly used to contrast photosynthesis, is vital for organisms to flourish on the dark seafloor where various reducing chemicals, such as H_2_, CH_4_, HS^−^, and Fe(II), are emitted from vent fluid and oxidized by O_2_ and NO_3_^−^ from seawater for microbial energy generation (Amend and Teske, 2005). In this case, the reductants are produced by rock-water interactions and magma degassing, whereas the oxidants are produced by photosynthesis-based biogeochemical processes. Microbial life dependent on oxidants that are produced independent of photosynthesis might be analogous to the microbial life of the primitive ocean before the emergence of photosynthetic life. Thus, deep-sea hydrothermal vents are considered a window for the subvent biosphere where photosynthetic products are negligible (Deming and Baross, 1993). The life search was extended from deep-sea hydrothermal vents at mid-oceanic ridges to ridge flanks associated with thermally and/or hydrologically driven fluid circulations (Edwards et al., 2011). As the thermal limit of life extends deeper in the oceanic crust as ridges cool down with time during spreading, microbial ecosystems are expected to be found below the seafloor where the maximum optimal growth temperature reaches the 120°C isotherm at a depth of 6km (Heberling et al., 2010; Heuer et al., 2020).

Similar to oceanic settings, intensive investigations on the microbial life of hot springs and deep groundwater sources on land have been conducted. The subsurface lithoautotrophic microbial ecosystem (SLiME), which does not depend on phototrophic organisms, was first discovered in a deep aquifer sustained by cretaceous flood basalt (Stevens and McKinley, 1995). The microbial ecosystem harvests energy by oxidizing H_2_ produced by *in situ* reactions between groundwater and olivine and pyroxene group minerals. Given that low-temperature dissolution rates of olivine and pyroxene group minerals are slow under neutral to slightly alkaline pH conditions that prevail in the deep subsurface, elevated temperatures resulting from hydrothermal activities appear to be favorable for SLiMEs owing to the accelerated rates of mineral-water reactions, as exemplified by the dominance of methanogenic archaea in hot springs (Chapelle et al., 2002). SLiMEs are important not only to understand primitive microbial life before the emergence of phototrophic organisms but also to search for extraterrestrial life on Mars, the surface of which has been harsh for phototrophic organisms for 3billion years (Onstott et al., 2019). Without photosynthesis, energy sources and fluxes derived from magma degassing, water-rock interactions, and radiolysis are essential.

## Technologies Unveiling Microbial Life in the Rocky Biosphere

It is well known that ~98% of microorganisms in nature are unculturable for isolation (Ward et al., 1990; Wade, 2002); thus, culture-independent techniques to identify natural microbial communities are necessary. PCR amplification of small subunits of rRNA and subsequent DNA sequencing has revealed the domain-level novelty of microorganisms named archaea (Woese, 1987). Diverse archaeal and bacterial groups from terrestrial hot springs have been identified (Barns et al., 1994; Hugenholtz et al., 1998), and thermophilic to hyperthermophilic groups are deep branching in the universal tree bases on rRNA sequences. As the primitive Earth appears to have been hot soon after the magma ocean cooled, hydrothermal vents are considered life’s cradle, and deep-branching prokaryotes are considered to conserve primitive features inherited from the common universal ancestor (Pace, 1991; Russell, 2021).

With molecular biological tools, the search for microbial life has been extended from hot springs to underground by drilling. Because it is critical to distinguish microbial contamination introduced from the drilling fluid, physically analogous tracers, such as fluorescent microspheres, along with dissolved chemical tracers have been applied to monitor the disturbance of geochemical and microbiological properties in the subsurface (Griffin et al., 2018). In scientific ocean drilling projects, an improved chemical tracer method with a volatile compound has been applied (Smith et al., 2000). Although microbiological investigations have been intensively conducted for deeply buried marine sediments (Inagaki et al., 2015; Orsi, 2018; Heuer et al., 2020) and terrestrial sedimentary rocks (Bagnoud et al., 2016; Daly et al., 2016; Hernsdorf et al., 2017; Magnabosco et al., 2018; Probst et al., 2018), underlying basement rocks remain poorly explored. This is mainly due to the technical difficulty in drilling the rocks (Michibayashi et al., 2019). In basement rocks, rock fractures and voids are directly connected from the core exterior, making it difficult to avoid severe contamination. In contrast, the contamination of unconsolidated sediments and sedimentary rocks can be easily avoided by removing the contaminated core exterior. Alternatively, pristine groundwater sources outflowing spontaneously from basement rocks through boreholes drilled from underground facilities at mines have been intensively studied for consolidated volcanic and sedimentary rocks. In an outstanding study conducted at a gold mine in South Africa, a “single-species ecosystem” containing a population of chemolithoautotrophic Firmicutes, “*Candidatus* Desulforudis audaxviator,” was found in 3-km-deep groundwater using chemical compounds produced by radiolytic reactions. The metabolic potential of the bacterial species, capable of performing sulfate reduction and fixing nitrogen and carbon, was supported by a metagenomic analysis of the groundwater sample, by which the complete genome was reconstructed (Chivian et al., 2008).

As demonstrated in the single-species ecosystem, near-complete to complete genomes were first reconstructed from natural microbial communities represented by high biomass and low species richness (Tyson et al., 2004). Later advances in DNA sequencing and bioinformatics enabled the genome-resolved metagenomic analysis of diverse microbial populations (Rinke et al., 2013). By assembling high-throughput sequences, high-quality genome sequences were obtained from candidate bacterial phyla without cultured representatives (Wrighton et al., 2012). Previously, their existence was only known from their 16S rRNA gene sequences (Hugenholtz et al., 1998).

**Figure 1:**
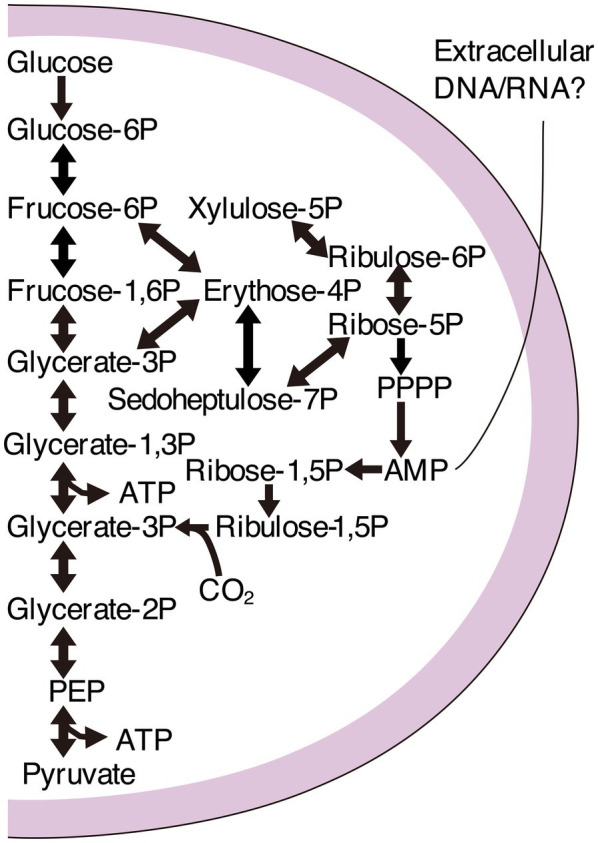
Pathway map of the carbon metabolism commonly found in Candidate Phyla Radiation (CPR) and Pacearchaeota. Glycolysis, the nucleotide salvage pathway, and the nonoxidative pentose phosphate pathway are shown to uptake extracellular nucleic acids.

Genomic characterizations have revealed that large bacterial lineages have relatively small genomes and cell sizes. They cannot often synthesize lipids, amino acids, and nucleotides (Brown et al., 2015; Luef et al., 2015). The bacterial group forming a monophyletic clade in phylogenetic trees based on ribosomal protein sequences is called the Candidate Phyla Radiation (CPR; Hug et al., 2016). Like CPR, the superphylum DPANN (Diapherotrites, Parvarchaeota, Aenigmarchaeota, Nanohaloarchaeota, and Nanoarchaeota) is a phylogenetically diverse group of archaea. DPANN represents a substantial fraction of archaeal diversity with small cell sizes and genomes, often lacking many core metabolic functions, such as pathways for synthesizing nucleotides, amino acids, and lipids (Castelle et al., 2015, 2018; Castelle and Banfield, 2018). CPR and DPANN may have diverged early from other bacteria and archaea (Castelle and Banfield, 2018) with metabolic platforms consistent with anaerobic conditions that were prevalent on the early Earth (Schönheit et al., 2016; Castelle and Banfield, 2018; Méheust et al., 2019). In particular, glycolysis, the nucleotide salvage pathway, and the nonoxidative pentose phosphate pathway are likely ancient and conserved among CPR and DPANN (Jaffe et al., 2020; Figure 1).

**Figure 2:**
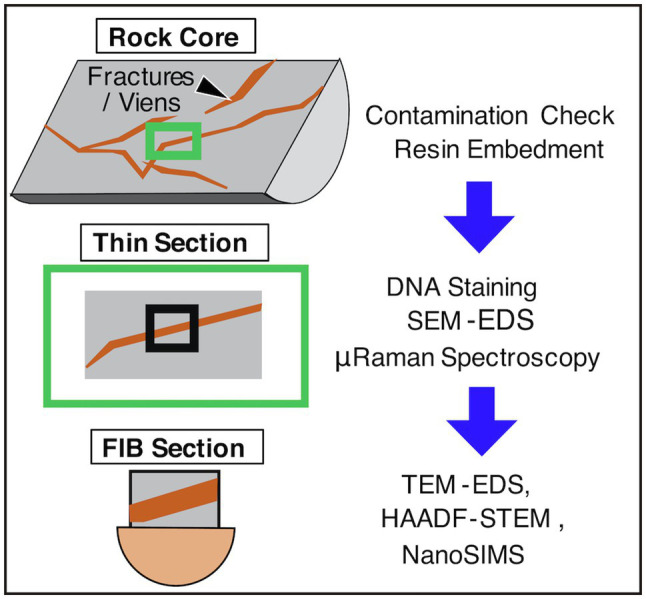
Flow chart of rock core characterizations. SEM, scanning electron microscopy; EDS, energy-dispersive X-ray spectroscopy; FIB, focused ion beam; TEM, transmission electron microscopy; NanoSIMS, nanoscale secondary ion mass spectrometry; HAADF-STEM, high-angle annular dark field-scanning transmission electron microscopy.

Apart from the advances in genome analysis and in addition to the usage of an ultraclean room for microbial cell counting (Heuer et al., 2020), microbial cell visualization in unconsolidated sediments has been technically improved using a high-sensitivity DNA dye called SYBR-Green I along with cell separation from sediment matrices by flow cytometry (Morono et al., 2013), in addition to the usage of an ultraclean room to count microbial cells (Heuer et al., 2020). Furthermore, volcanic rocks and drill cores, which are thought to be the most challenging environments for microbial characterization, have also been approached using a new method (Sueoka et al., 2019). In the new method, volcanic rock cores with fractures were embedded in resin and subsequently sliced into thin sections to visualize the rock interior. As the resin, called LR White, is hydrophilic, SYBR-Green I penetrated the solidified resin, and microbial cells in the rock’s interior were stained and visualized by fluorescence microscopy (Figure 2).

Microbial cells in thin sections are characterized by submicron-scale spectroscopic and spectrometric analyses and nanoscale mineralogical identification (Yamashita et al., 2019; Suzuki et al., 2020; Figure 2). For thin sections with a thickness of ~100μm, μ-Raman spectroscopy is used to clarify the distributions of functional groups in organic compounds and minerals at a beam diameter of ~1μm. Scanning electron microscopy coupled with energy-dispersive spectroscopy (SEM-EDS) is used to obtain the submicron-scale compositions of minerals. To obtain submicron-scale distributions of carbon, nitrogen, sulfur, and phosphorous in microbial cells by nanoscale secondary ion mass spectrometry (NanoSIMS), it is necessary to fabricate thin sections with a thickness of ~3μm using a focused ion beam (FIB). To identify minerals around microbial cells, FIB sections need to be thinned down to a thickness of 100nm for transmission electron microscopy EDS (TEM-EDS) and high-angle annular dark field-scanning transmission electron microscopy (HAADF-STEM). The higher the spatial resolution, the more damaged the sample. Nevertheless, high-resolution analytical data are crucial to conclude that signals stained by SYBR-Green I are derived from microbial cells.

For microbiological characterizations of rock core samples, it is necessary to evaluate microbial contamination during drilling (Li et al., 2020; Suzuki et al., 2020). For the new method, a procedure initially developed by Lever et al. (2006) was applied (Suzuki et al., 2020; Figure 2). Using a hammer and chisel, rock cores were separated into core exterior and core interior, where fluorescent microspheres were introduced from drilling fluid. After the collection of the microspheres by ringing the core portions with artificial seawater, the microspheres were counted by microscopy. The exterior of the core sample was slightly flamed to remove any surface contamination. Finally, the core interior was separated from the core exterior and then subjected to microbiological characterizations. To clarify the occurrence of microbial contamination, 16S rRNA gene amplicon analysis was performed for the core interior and controls, including the contaminated exterior, drilling fluid, bottom seawater, and experimental controls (Suzuki et al., 2020).

## Biogeochemical Processes Apart From Photosynthesis

Around deep-sea hydrothermal vents, organisms flourish without photosynthetic organics and with and without the supply of oxidants derived from oxygenated photosynthesis (Hügler and Sievert, 2011). Sunlight can physically reach up to 200m in the ocean (Madigan et al., 2008). In the water column surface, photosynthetic products sink or are circulated through ocean currents to reach the seafloor. In deep-sea environments, metabolic activities using photosynthetic organics are limited. Instead of heterotrophy, chemolithoautotrophy based on ammonia, a decomposition product of photosynthetic organics, is mediated by archaea belonging to the phylum Thaumarchaeota (Brochier-Armanet et al., 2008). Hereafter, we list organism names according to the National Center for Biotechnology Information taxonomy (Federhen, 2012). Ammonia-oxidizing archaea are among the most abundant organisms on Earth. Nitrite oxidation by Proteobacteria, Nitrospira, and Nitrospina is also important for chemolithoautotrophy in meso- and bathypelagic zones (Hügler and Sievert, 2011). In the deep seafloor region, >99% of photosynthetic organics are degraded in the water column (Van Cappellen, 2003). Hence, in subseafloor sediments, the rates of metabolic reactions involving the oxidation of photosynthetic organics are substantially low (Morono et al., 2020). Based on differences in the concentrations of electron acceptors used to oxidize photosynthetic organics in deep marine sediments at different depths, it can be ascertained that microbial cells have slow metabolism (Wang et al., 2008) and rarely divide, with energy consumption up to six orders of magnitude lower than that of cells living in surface habitats (Price and Sowers, 2004; Hoehler and Jørgensen, 2013).

Similarly, microorganisms have also been reported to slowly metabolize recalcitrant organics in terrestrial aquifers. In these cases, deep groundwater samples were obtained from wells drilled to track groundwater flow (Chapelle and Lovley, 1990; Phelps et al., 1994). By determining the ages of the groundwater samples along with changes in the concentrations of dissolved carbonate species and electron acceptors, the metabolic rates of the oxidation of organics freshly supplied from modern photosynthetic activities and deeply buried after the deposition of sediment particles were determined. In deserts where freshly supplied organics from vegetation are inadequate, O_2_ deeply penetrates the subsurface environment without being consumed by heterotrophic microorganisms (Winograd and Robertson, 1982). Additionally, the extents of photosynthetic products supplied in deep aquifers vary by the spatiotemporal scales of groundwater flow previously defined as local, intermediate, and regional flow systems (Lovley and Chapelle, 1995).

## Basics of the Deep Rocky Biosphere

Onstott et al. (2019) defined rock-hosted life as “the existence of which is critically dependent upon physicochemical processes within the host rock.” Compared with the surface photosphere, which gains abundant energy from sunlight, rock-based microenvironments have different energetic advantages. There appear to be two major processes offering energetic advantages: interactions between water and mafic minerals, such as olivine and pyroxene group minerals, and radiolytic reactions (Figure 2). The latter process was demonstrated in the study in South Africa on gold mine groundwater colonized by a single bacterial species. In gold- and uranium-enriched archaeal formations, the radiolysis of water produces H_2_ and reactive oxygen species, such as H_2_O_2_ (Lin et al., 2005; Figure 2). The reaction of H_2_O_2_ with sulfide minerals, such as pyrite (FeS_2_), leads to the formation of sulfate (the reaction product), which is used as an electron acceptor (Lefticariu et al., 2006). The sulfate reducer is classified within the phylum Firmicutes, many members of which form spores to survive in harsh conditions (Madigan et al., 2008). In the reconstructed genome, genes involved in carbon and nitrogen fixation pathways are fully encoded.

In mafic and ultramafic rocks, such as basalt and peridotite, olivine and pyroxene group minerals reacted with water to produce not only H_2_ but also hydrocarbons (Charlou et al., 2002; Proskurowski et al., 2008; Figure 2). This process, known as serpentinization, is accompanied by the formation of serpentine group minerals. One of the remarkable vent fields associated with water interactions with peridotite is the Lost City, an off-axis hydrothermal vent field near the Mid-Atlantic Ridge (Kelley et al., 2001). White chimneys mainly composed of carbonate minerals formed from alkaline vent fluids host microbial communities on the seafloor. The chimney interior is dominated by a single phylotype of archaea from the order Methanosarcinales (Schrenk et al., 2004), whereas anaerobic methane-oxidizing archaea are found in the chimney exterior (Brazelton et al., 2006). The methanogenic members of the order Methanosarcinales use acetate or methyl compounds, such as methylamines and methylsulfates (Kendall and Boone, 2006). These substrates appear to be products of serpentinization. Geological settings where rock-hosted life has been demonstrated, such as in the South African gold mine aquifer and the Lost City vent chimneys, are rather exceptional in the present day. Geological formations that are currently prevalent are composed of felsic and mafic rocks in the terrestrial and oceanic crusts, respectively.

## The Oceanic Crust Biosphere

**Figure 3:**
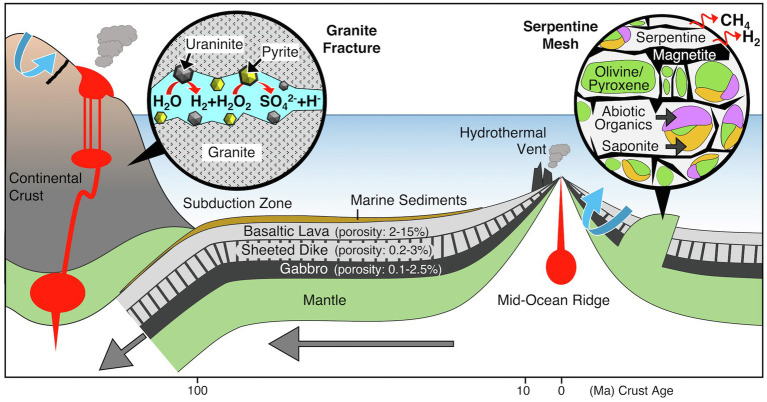
Geologic and tectonic characteristics of the deep rocky biosphere. Mineralogical and geochemical characteristics of habitats for rock-hosted life are shown in circular frames. Blue arrows indicate the circulation of hydrothermal fluid.

**Figure 4:**
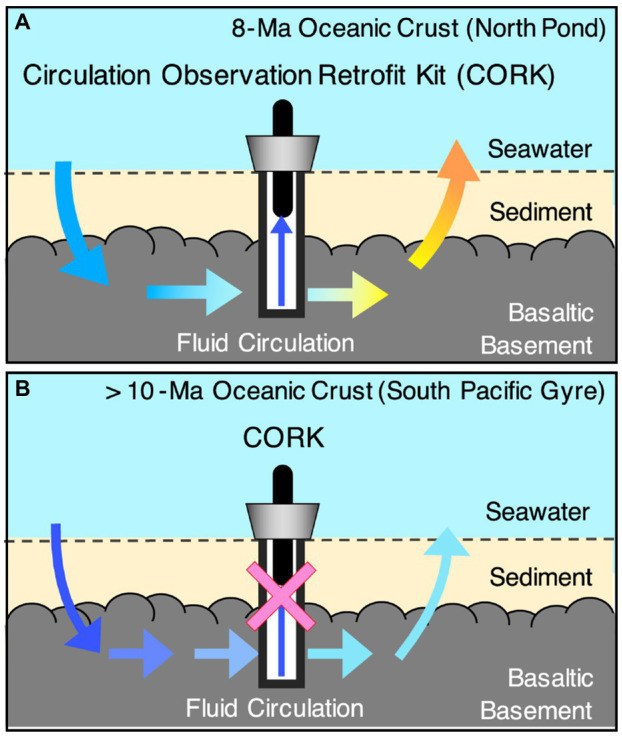
Schematic of the flow regime and sampling of crustal fluid at North Pond site **(A)**. Schematic of the flow regime and the lack of retrievable crustal fluid at the South Pacific sites **(B)**. Blue and red arrows in **A** and **B** indicates abundant substrate supplies from seawater and basalt rocks, respectively.

The oceanic crust is formed at mid-oceanic ridges by the cooling of basaltic magma (Figure 3). The upper oceanic crust is composed of basaltic lavas with high porosity (2–15%) that resulted from rapid cooling by seawater, whereas the porosity of the lower oceanic crust is much lower than that of the upper oceanic crust: 0.2–3% in sheeted dikes and 0.1–2.5% in gabbro (Heberling et al., 2010). Given the porosity of the oceanic crust below the 120°C isotherm (~10^9^km^3^), Heberling et al. (2010) estimated that the oceanic crust biomass is equivalent to the prokaryotic biomass in the entire ocean (~10^29^ cells; Bar-On et al., 2018). At mid-ocean ridges, high-temperature basalt-seawater reactions provide substantial energy for sustaining life (Bach and Edwards, 2003). On the ridge flank, the circulation of crustal fluid is hydrothermally and/or hydrologically driven within the basaltic lava overburdened with sediments (Sclater et al., 1980; Figure 4). The portion of basaltic lava underneath the sediment cover is referred to as the basaltic basement. Previous studies at 3.5- and 8-million-year-old (Ma) ridge flank systems demonstrated that these young crustal aquifers harbor anaerobic thermophiles and aerobic mesophiles contributing to carbon and sulfur cycling, respectively (Cowen et al., 2003; Lever et al., 2013; Orcutt et al., 2013; Zhang et al., 2016). After rock fractures are filled with secondary minerals, the intensities of fluid circulation and basalt-seawater reactions sharply decline along with increasing crustal age, especially after 10Ma (Jarrard, 2003; Figure 4). Although >90% of the ocean lithosphere of Earth comprises oceanic crust >10Ma (Müller et al., 2008) and microbial-like textures have been widely observed at the glassy margin of basaltic lava (Fisk et al., 1998; Staudigel et al., 2008), the existence of microbial life in the spatially vast crustal environment remains largely unknown, partly because of the technological and analytical challenges of exploring the igneous rock habitat through scientific drilling (Santelli et al., 2010).

It is technically difficult to collect pristine crustal fluid from basement igneous rocks *via* boreholes drilled from research vessels, unlike land-based subsurface investigations. Without geochemical information from the crustal fluid, the habitability of the rocky environment remains largely unknown. For basaltic basements without retrievable crustal fluids, 13-Ma, 33-Ma, and 104-Ma basaltic lavas have been drilled during the Integrated Ocean Drilling Program Expedition 329, which targeted life beneath the seafloor of the South Pacific Gyre (SPG; Figure 4). The SPG is an oceanic region where surface photosynthetic activity is exceedingly low (D’Hondt et al., 2015). This ultra-oligotrophic feature might favor microorganisms living independently from photosynthetic organics in the underlying basaltic basement (Morono et al., 2020). To understand the rocky biosphere in the basaltic basement, a new life-detection technique has been successfully developed for drilled rock cores combined with nanoscale mineralogical characterizations (Sueoka et al., 2019; Yamashita et al., 2019). Basalt fractures filled with clay and calcium carbonate minerals are associated with the formation of Fe- and Mg-smectite minerals that are compositionally and structurally similar to saponite and nontronite. Both smectite minerals are good indicators of low-temperature basalt-water interactions. The dense colonization of microbial cells has been directly visualized to exceed ~10^10^ cells/cm^3^, in spatial association with nontronite (Suzuki et al., 2020). More surprisingly, heterotrophic bacteria are dominant, as demonstrated by DNA sequencing and lipid analysis (Suzuki et al., 2020).

Genome-resolved metagenomic analysis of crustal fluids in the oceanic crust was conducted (Jungbluth et al., 2016; Tully et al., 2018). Metatranscriptomic analysis was combined with metagenomic analysis to examine metabolic gene expressions in cold crust fluids (Seyler et al., 2021). Metatranscriptomic analysis was also performed for drilled rock cores obtained 750m below the seafloor at Atlantis Bank, Indian Ocean, where Earth’s lower crust is exposed at the seafloor (Li et al., 2020).

The 8-Ma ridge flank system called North Pond on the Mid-Atlantic Ridge is one of the most extensively characterized oceanic crusts owing to data creation from time series metagenomic and metatranscriptomic analyses and bulk and single-cell metabolic rate measurements (Trembath-Reichert et al., 2021). Despite the lack of inorganic electron donors, carbon fixation transcripts are associated with the highest rates of bicarbonate incorporation. Metagenomic analysis supports carbon fixation pathways linked to sulfide and thiosulfate oxidation, along with a mixotrophic lifestyle represented by numerous extracellular protease and carbohydrate catabolism genes. Metatranscriptomic analysis has also revealed that organotrophic processes are predominant in subseafloor ultramafic and gabbroic rocks cored at the Atlantis Bank (Li et al., 2020), which is consistent with the results from gabbroic rocks cored at the Atlantis Massif (Mason et al., 2010). These results are consistent with those from the SPG, where heterotrophic bacteria were found to be predominant in 33- and 104-Ma basaltic basements (Suzuki et al., 2020).

Trembath-Reichert et al. (2021) hypothesized that both organic and inorganic carbon substrates are assimilated in the rocky subseafloor, which reflects the optimization of microbial communities to energy-limited conditions. Deep-sea dissolved organics may be largely oxidized for energy by organotrophic microorganisms, whereas bicarbonate may serve as a supplementary carbon source.

## The Continental Crust Biosphere

Considering the porosity of the continental crust below the 120°C isotherm (~10^9^km^3^; Magnabosco et al., 2018), the prokaryotic biomass in this region is estimated to be equivalent to that in the ocean. The surface of the continental crust is covered with shales (~50%), sandstone (~15%), and granite (15%; Leopold et al., 2020). Shales are originally formed at the seafloor, where photosynthetic organics are deposited. Sandstone is formed by river and ocean waves at near-surface settings. The biosphere minimally depending on photosynthetic products could be hosted in deep granitic rocks, given that photosynthetic organics and oxidants are consumed at the shallow subsurface. Within the continental crust, granitic rocks are formed by the cooling of intruded felsic magma. As a result, organics are initially absent. Given the low content of olivine and pyroxene group minerals that effectively produce H_2_ (Bach, 2016), H_2_ production by serpentinization reactions is negligible in granitic rocks in low-temperature settings. As granite rocks are enriched with uranium (Langmuir, 1997), H_2_ production by the reaction of water with radiation from radionuclides, such as ^238^U or ^40^K, is associated with radiolysis (Lin et al., 2005). In granitic rocks, abiotic methane from magmatic processes is ubiquitous long after formation (Etiope and Sherwood Lollar, 2013; Kietäväinen and Purkamo, 2015). However, it remains unknown whether methane can serve as a major energy source in the deep granitic environment.

Microbiological investigations of the underground facilities constructed in the granitic basements of the Canadian and Scandinavian Shields have been conducted (Jain et al., 1997; Pedersen, 2000; Pedersen et al., 2014). Granitic basement widely distributed in the Scandinavian Shield has undergone seawater intrusion into the deep aquifer, where the last deglaciation triggered a rise in the sea level. As the intruded seawater is enriched with 0.1–50mM dissolved organic carbon (DOC), the H_2_ required for the microbial reduction of CO_2_ likely originates from the microbial fermentation of organics produced by photosynthesis (Pedersen, 2012). In the seawater-dominated granitic basement of the Olkiluoto island in Finland, sulfate is microbiologically reduced near the surface and is depleted at ~500mbgl, where hydraulic conductivities range from 3.0×10^−7^ to 1.5×10^−11^m/s (Pedersen et al., 2008; Vaittinen et al., 2011). The deeper basement with low hydraulic conductivities (<1.5×10^−11^m/s) was associated with the increase in CH_4_ concentration and with the decrease in dissolved inorganic carbon. In contrast, the seawater-dominated granitic basement for the final disposal of high-level nuclear wastes in Sweden is characterized by high hydraulic conductivities ranging from ~10^−5^ to ~10^−8^m/s (Laaksoharju et al., 2008) and high levels of sulfate (>0.2mM) at depths of 112–978mbgl (Hallbeck and Pedersen, 2012). Recently, ^13^C-depleted carbonate was found in deep granite fractures associated with modern seawater intrusion, which suggests that methane is an important energy source at the Swedish candidate site (Drake et al., 2015).

Although the Canadian and Scandinavian Shields are accompanied by ancient and modern intrusions of photosynthetic organics, granitic basements are generally percolated by meteoric water with residual organics recently recharged from shallow groundwater. To explore the granitic biosphere, 69-Ma granite rocks were drilled horizontally from a 300-m deep underground tunnel at the Mizunami Underground Research Laboratory (URL) in Gifu Prefecture, central Japan. Regardless of the vegetation- and soil-related surface processes, the DOC levels are low in the deep granitic basement, with a negligible supply of H_2_ (Suzuki et al., 2014). Sulfate and CO_2_ are the dominant oxidants. The range of hydraulic conductivity of ~10^−11^m/s is the hydrological threshold for the biogeochemical shift from sulfidic to nonsulfidic conditions in the granitic basement (Ino et al., 2016, 2018). A cretaceous granitic basement was horizontally drilled from an underground tunnel at the Grimsel Test Site on the Swiss Alps. Freshwater-dominated groundwater was collected with the levels of sulfate (51.6–176.8μM) and DOC (22.4–82.7μM) similar to those observed in the highly fractured domain of the Mizunami URL (Konno et al., 2013). Notably, sulfate was also detected in the range of hydraulic conductivities of 1.4×10^−9^ to 3.3×10^−8^m/s.

Genome-resolved metagenomic and metatranscriptomic analyses have been conducted for 171- to 448-m deep groundwater samples from the Äspö Hard Rock Laboratory, where Baltic Sea-influenced water with a residence time of <20years, defined as “modern marine,” shifts with depth to form “old saline” groundwater, with a residence time of thousands of years (Wu et al., 2016; Lopez-Fernandez et al., 2018). Proteobacteria, Candidate divisions OD1 and OP3, currently classified as *Candidatus* Parcubacteria (a major phyla of CPR), and *Candidatus* Omnitrophica, unclassified archaea and unclassified bacteria, are dominant in the groundwater samples. Metabolic activities are estimated to be heterotrophic in “modern marine,” whereas the proportions of H_2_-depedent chemoautotrophs are higher in “old saline” than in “modern marine.” Metagenomic and metaproteomic analyses of 366.7–383.5-m deep groundwater samples highlight the dominance of Deltaproteobacteria and the importance of sulfur cycling by phylogenetically and physiologically diverse microbial populations (Bell et al., 2020). In 300-m deep groundwater at the Mizunami URL, genome-resolved metagenomic analysis indicates that anaerobic methane-oxidizing archaea are harvesting energy from magmatic methane under energy-limited conditions (Ino et al., 2018). In addition, *Candidatus* Parcubacteria and *Candidatus* Omnitrophica appear to be dominant in the deep granite biosphere flushed with meteoric water.

## Future Prospects of Deep Rocky Biosphere Research

In addition to the dissolved organic matter in seawater that flows through fractures and veins (Walter et al., 2018), organic matter appears to be abiotically synthesized during serpentinization at a depth of 173 mbsf in the Atlantis Massif, where amino acid production is associated with saponite formation in the gabbroic basement (Ménez et al., 2018). Similarly, saponite-filled fractures are enriched with organic matter in the 100-Ma basaltic basement, where microbial cells are densely colonized (Sueoka et al., 2019). Given that saponite is a smectite group mineral with a large surface area to adsorb dissolved organics (Cuadros, 2017), the roles of smectite and other fine-grained minerals in concentrating organics need to be investigated in various geological and tectonic settings.

Apart from concentrating organic matter, the roles of minerals in catalyzing the prebiotic synthesis of the building blocks of life from simple reduced chemicals and their polymerization have been hypothesized to give rise to the cradle of life (Hazen et al., 2012; Russell, 2021). In terrestrial hot springs, amorphous silica, smectite and other clay minerals, metal sulfides, and their assemblages are essential for protection against ultraviolet light (Mulkidjanian et al., 2012). Similar mineral assemblages are also hypothesized to occur in deep-sea hydrothermal vents (Martin et al., 2008). Although modern hydrothermal vents are exposed to oxygenated conditions, the rock interior associated with fluid emanation could be analogous to anoxic geothermal fields. Even after the cessation of fluid venting, mineral assemblages in rocks could sustain life-emerging processes under anoxic conditions. In contrast to microbial life derived from hydrothermal fluids, whether microbial colonization is associated with specific minerals in the rock interior with and without fluid venting remains largely unexplored. Eco-physiological features of rock-hosted life in life-emerging settings may constrain how life could thrive in primitive habitats on Earth.

In the solar system, rock-hosted life may be present beyond Earth. It is known that deep-sea hydrothermal activities occur in the subocean on Saturn’s icy moon Enceladus (Glein and Zolotov, 2020). Plumes erupted from cracks in the ice have been analyzed to infer that the subocean is salty (Postberg et al., 2009) with silica particles indicative of rock-water interactions (Hsu et al., 2015). The subocean silicate crusts of Europa and Enceladus may host extant life in low-temperature groundwater/hydrothermal systems. On Mars, Fe- and Mg-smectite mineral formation from basaltic rocks had occurred ubiquitous at the surface and in the subsurface until ~3 billion years ago (Ehlmann et al., 2011). Currently, the surface is cold and dry under high vacuum conditions with methane emission from the subsurface into the atmosphere (Webster et al., 2018). Given the subsurface presence of liquid water (Orosei et al., 2019), extant rock-hosted life and/or their biosignatures will be discovered on Mars (Onstott et al., 2019). Outside the solar system, Lingam and Loeb (2021) reported that worlds with the capacity to harbor deep biospheres might be up to ~100 times more abundant than those that can sustain phototrophy-based surface biospheres.

## Conclusion

In this study, our current understanding of the rocky biosphere was documented to emphasize that microorganisms can harvest inorganic and organic energy sources independently from photosynthesis. Omics-based approaches and nanosolid characterizations have begun to unveil metabolic pathways suitable for thriving with mineral assemblages prevalent on early Earth and other planetary bodies potentially harboring extant life.

## Author Contributions

HT collaborated with the corresponding author (YS) in the construction of manuscript. MK produced figures. All authors contributed to the article and approved the submitted version.

## Funding

This work was supported by JSPS KAKENHI Grant Number 20H03319. YS was partly funded by the Astrobiology Center Program of National Institutes of Natural Sciences (NINS; GRAB311023).

## Conflict of Interest

The authors declare that the research was conducted in the absence of any commercial or financial relationships that could be construed as a potential conflict of interest.

## Publisher’s Note

All claims expressed in this article are solely those of the authors and do not necessarily represent those of their affiliated organizations, or those of the publisher, the editors and the reviewers. Any product that may be evaluated in this article, or claim that may be made by its manufacturer, is not guaranteed or endorsed by the publisher.

## Abbreviations

CPR: Candidate Phyla Radiation
DOC: Dissolved organic carbon
DPANN: Diapherotrites, Parvarchaeota, Aenigmarchaeota, Nanohaloarchaeota, Nanoarchaeota
FIB: Focused ion beam
Ma: Million-year-old
PCR: Polymerase chain reaction
SLiME: Ssubsurface lithoautotrophic microbial ecosystem
SPG: South Pacific Gyre
URL: Underground Research Laboratory
